# Formalizing the law of diminishing returns in metabolic networks using an electrical analogy

**DOI:** 10.1098/rsos.240165

**Published:** 2024-10-02

**Authors:** Marianyela Petrizzelli, Charlotte Coton, Dominique de Vienne

**Affiliations:** ^1^ Université Paris-Saclay, Gif-sur-Yvette 91190, France

**Keywords:** diminishing returns, metabolic flux, electrical circuit, robustness

## Abstract

The way biological systems respond to changes in parameter values caused by mutations is a key issue in evolution and quantitative genetics, as it affects fundamental aspects such as adaptation, selective neutrality, robustness, optimality, evolutionary equilibria, etc. We address this question using the enzyme–flux relationship in a metabolic network as a model of the genotype–phenotype relationship. The lack of a suitable mathematical tool from biochemical theory to investigate this relationship led us to use an analogy between electrical circuits and metabolic networks with uni–uni reactions. We show that a behaviour of diminishing returns, which is commonly observed at various phenotypic levels, is inevitable, irrespective of the complexity of the system. We also present a possible generalization to metabolic networks with both uni–uni and bi–bi reactions.

## 1. Introduction

As reviewed and discussed in various papers [1,2], the genotype–phenotype relationship, as well as the relationship between adjacent or distant phenotypic levels, often seems to follow a law of diminishing returns: as the value of a given parameter increases, the gain in value of the phenotypic output becomes increasingly smaller and the curve reaches a horizontal asymptote. This type of response has been observed at various phenotypic levels. For instance, the relationship between factors of the transcriptional and/or translational machinery and (i) gene expression levels [3], (ii) protein synthesis rates [4], (iii) morphological traits [5], and (iv) fitness [6–8] is commonly concave.

The archetypal behaviour of diminishing returns is displayed by the relationship between enzyme activity and flux [9,10]. This relationship has been comprehensively analysed during the past 50 years within the framework of metabolic control analysis (MCA) [11,12]. However, the MCA formalism has been mainly developed for linear pathways and not for networks. The way the total flux through a network changes in response to variations in enzyme parameters is a central question in quantitative and evolutionary genetics. In principle, this question can be addressed with systems of ordinary differential equations. However, most of the time we do not have a sufficient knowledge of the *in vivo* parameter values, and furthermore, this approach is not informative regarding possible general behaviours: beyond the specific responses of particular networks, is there a shape of the enzyme–flux relationship that would be *qualitatively* valid for a majority of situations? Intuitively, a behaviour of diminishing returns makes sense: irrespective of the complexity of a network, the effect of increasing a particular parameter value is limited by the fixed values of the other parameters. The purpose of this study is to examine the validity of this idea.

## 2. An electrical analogy for metabolism

### 2.1. The value of analogies

We did not find a general mathematical tool within the framework of biochemical theory to investigate the question of diminishing returns in metabolic networks. Therefore, we decided to use an approach from outside biology, namely mathematics applied to electrical circuits.

Analogies in science are based on shared properties and enable the illustration or clarification of a system’s attributes based on the attributes of another system. Indeed, electricity, mechanics, hydraulics, electrokinetics, electromagnetism, thermodynamics and heat transfer have similar equations describing the transfer of matter or energy, and thus they can illuminate each other [13]. In biology, the most commonly used analogy to investigate metabolic fluxes, but also to a lesser extent transcription networks [14], signalling networks [15], regulatory networks [16], is electricity, under more or less sophisticated forms. To our knowledge, the first analogy between metabolism and electricity was proposed in the context of genetics as a means to illustrate a biochemical model of heterosis [17]. In this analogy, a chain of enzymes was likened to a series of pairs of resistors in parallel, each pair corresponding to the two alleles of each enzyme-coding gene. Along the same line, Yi & Dean [18] equated the flux in the lactose pathway of *Escherichia coli* to an electrical current and the associated enzyme activities to conductances. They used this analogy to illustrate the use of adaptive landscapes for predicting the distribution of fitness effects. In the context of flux balance analysis (FBA), Segrè *et al*. [19] observed that the stoichiometric constraints in a metabolic network at a steady state are treated analogously to Kirchhoff’s first law for the balance of currents in electrical circuits. It was then shown that applying thermodynamic constraints that are analogous to Kirchhoff’s second law to FBA models restricts the allowable distribution of fluxes [20–22]. In addition, Price *et al*. [21] used the analogy of charging and draining a battery to interpret type I and type II extreme pathways. In a much more formal way, Ederer & Gilles [23,24] developed the thermodynamic-kinetic modelling (TKM) to describe biochemical networks in the language of electrical engineering, thus highlighting the relationship between concentrations and thermodynamic forces. Liebermeister *et al*. [25] highlighted the equivalence between the TKM rate laws and their so-called modular rate laws. Cardelli *et al*. [26] showed how to convert a linear electric circuit to a chemical reaction network of the same functionality through systems of differential algebraic equations. Finally, this analogy was also used for teaching purposes, where the three major regulatory enzymes of the glycolytic pathway were compared with the three key parts of an electrical power generation system [16].

In this study, following [17] and [18], we consider that enzymes are analogous to resistors and metabolites are analogous to nodes, in networks of any topology. We fully develop this theory for networks of uni–uni Michaelis–Menten reactions, then provide elements for dealing with networks that comprise both uni–uni and bi–bi reactions.

### 2.2. Formalizing the analogy

In electricity, the current across a dipole is written as:


(2.1) $$I=\frac{U}{R},$$

where U is the potential difference and R the resistance. The ratio 1/R is the conductance of the dipole.

In enzymology, the rate of a uni–uni reaction catalysed by a Michaelian enzyme that is far from saturation is written as [9,27]:


(2.2) $$v\approx [E]\frac{k_{\text{cat}}}{K_{\text{M}}}\;\;\left(X_{\text{S}}-\frac{X_{\text{P}}}{K_{\text{eq}}}\right),$$

where [E], $k_{\text{cat}}$ and $K_{\text{M}}$ are, respectively, the concentration, the catalytic constant and the Michaelis constant of the enzyme, $X_{\text{S}}$ and $X_{\text{P}}$ are, respectively, the concentration of the substrate and concentration of the product of the reaction and $K_{\text{eq}}$ is the equilibrium constant of the reaction.

If we compare the forms of equations (2.1) and (2.2) we see that the reaction rate v is analogous to the electrical current I, the enzyme efficiency $F=[E]\;k_{\text{cat}}/K_{\text{M}}$ is analogous to the conductance 1/R and the difference $X_{\text{S}}-X_{\text{P}}/K_{\text{eq}}$ is analogous to the potential difference U.

The total flux through a metabolic network of any complexity is dependent on the enzyme efficiencies and the topology of the network, in the same way that the total current through an electrical circuit is dependent on the conductances and the topology of the circuit.

### 2.3. The concept of equivalent conductance

An important characteristic of an electrical circuit is its *equivalent resistance*, $R_{\text{E}}$, defined as the resistance of a single resistor that, if it replaced all resistors in the circuit, would result in the same total current. Thus, the *equivalent conductance* of the circuit is:


$$\sigma_{\text{E}}=\frac{1}{R_{\text{E}}}.$$

Because the total current through the circuit is


$$I=\sigma_{\text{E}}U,$$

where U is the potential difference at the circuit terminals, the equivalent conductance $\sigma_{\text{E}}$ is proportional to the total current I, 1/U being the proportionality constant.

In the same way, we can define the *equivalent enzyme efficiency*, $F_{\text{E}}$, of a metabolic network, whereby $X_{\text{S}}$ is metabolized into $X_{\text{P}}$ through a single pseudo-reaction. The total flux through the network is then written as:


(2.3) $$J=F_{\text{E}}\;\left(X_{S}-\frac{X_{P}}{K_{\text{E}}}\right),$$

where $K_{\text{E}}$ is the equivalent equilibrium constant that depends on all individual equilibrium constants.

The equivalent enzyme efficiency $F_{\text{E}}$ is proportional to the total metabolic flux J, $1/(X_{S}-X_{\text{P}}/K_{\text{E}})$ being the proportionality constant.

Therefore, characterizing the relationship between the conductance $\sigma_{ij}$ between nodes i and j and the equivalent conductance $\sigma_{\text{E}}\propto I$ in an electrical circuit of any complexity can help answer the question of the relationship between a particular enzyme efficiency $F_{ij}$ and the equivalent efficiency $F_{E}\propto J$ in a metabolic network of any complexity.

### 2.4. Simple circuits

**Figure 1.**
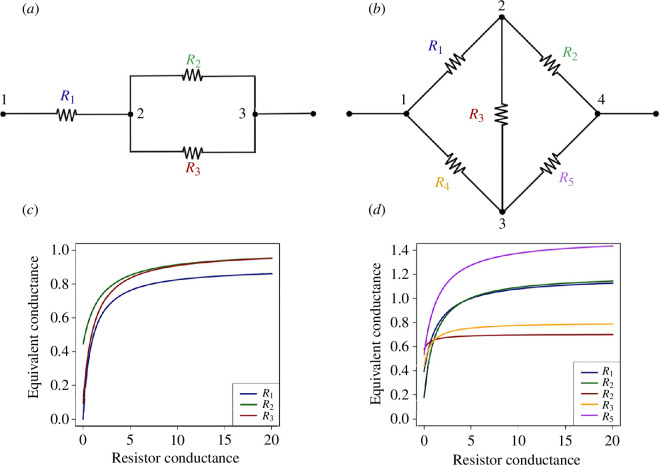
Two basic types of electrical circuits. (*a*) A circuit with resistors exclusively in series and in parallel. (*b*) A Wheatstone bridge. (*c*) Relationship between the equivalent conductance $\sigma_{\text{E}}$ and the individual conductances in circuit (*a*) (same colour code as in (*a*)). For each curve, one conductance increased from 0 to 20, the other conductances being fixed. The fixed conductance values are $1/R_{1}=1$, $1/R_{2}=10$ and $1/R_{3}=1.25$. (*d*) Relationship between the equivalent conductance $\sigma_{\text{E}}$ and the individual conductances in the Wheatstone bridge in (*b*). The fixed conductance values are $1/R_{1}=0.43$, $1/R_{2}=1$, $1/R_{3}=1.25$, $1/R_{4}=1$ and $1/R_{5}=5$.


If the resistors in the electrical circuit are exclusively in series and/or in parallel, the equivalent resistance and the equivalent conductance can be easily calculated by applying the rule of additivity for resistances and conductances, respectively. For instance, the circuit in figure 1*a* has resistor R_1_ in series with a bypass loop containing resistors R_2_ and R_3_ in parallel. Summing the conductances $\sigma_{2}$ and $\sigma_{3}$ of R_2_ and R_3_, respectively, then summing the resistances $1/\sigma_{1}$ and $1/(\sigma_{2}+\sigma_{3})$, we get

$$\sigma_{\text{E}}=\frac{\sigma_{1}(\sigma_{2}+\sigma_{3})}{\sigma_{1}+\sigma_{2}+\sigma_{3}},$$

or, using a notation where the conductances are indexed according to the node numbers flanking each resistor (see figure 1*a*):


$$\sigma_{\text{E}}=\frac{\sigma_{12}\sigma_{23}}{\sigma_{12}+\sigma_{23}},$$

where $\sigma_{12}=\sigma_{1}$ and $\sigma_{23}=\sigma_{2}+\sigma_{3}$. All the conductances are positive, thus it is easy to show that the relationship between $\sigma_{\text{E}}$ and any of the conductances is a concave hyperbole (figure 1*c*).

This reduction method of successively grouping resistances can be applied to circuits of any size provided they contain only resistors in series and in parallel.

### 2.5. Complex circuits

For circuits that do not contain resistors exclusively in series and/or in parallel, the rule of additivity for resistances and conductances cannot be used directly. Consider for instance a Wheatstone bridge, which represents the simplest case of a complex circuit (figure 1*b*): it is easy to show that the additivity rule does not apply. More sophisticated techniques, such as the *nodal potential method* [28] or the *Delta-Y method* that relies on the Kennelly’s theorem [29], have to be used.

Generalizing these approaches, Kagan [28] showed that in an *n*-node circuit (n>2) of any topology, the relationship between $\sigma_{\text{E}}$ and $\sigma_{ij}$ is:


(2.4) $$\sigma_{\text{E}}=\frac{A\sigma_{ij}+B}{C\sigma_{ij}+D},$$

where A, B, C and D are constants that depend on the conductances of the circuit out of $\sigma_{ij}$.

For instance, the relationship between $\sigma_{\text{E}}$ and the conductances in a Wheatstone bridge is:


(2.5) $$\sigma_{\text{E}}=\frac{\sigma_{12}\sigma_{24}(\sigma_{23}+\sigma_{13}+\sigma_{34})+\sigma_{24}\sigma_{23}\sigma_{13}+\sigma_{12}\sigma_{23}\sigma_{34}+\sigma_{13}\sigma_{34}(\sigma_{12}+\sigma_{24}+\sigma_{23})}{\sigma_{23}(\sigma_{12}+\sigma_{24}+\sigma_{13}+\sigma_{34})+(\sigma_{12}+\sigma_{24})(\sigma_{13}+\sigma_{34})}.$$

If the variable conductance is, say, $\sigma_{12}$, we have:


$$\begin{array}{rl} A & =\sigma_{24}\sigma_{34}+(\sigma_{13}+\sigma_{23})(\sigma_{24}+\sigma_{34})\\ B & =\sigma_{13}(\sigma_{23}\sigma_{24}+\sigma_{24}\sigma_{34}+\sigma_{23}\sigma_{34})\\ C & =\sigma_{13}+\sigma_{23}+\sigma_{34}\\ D & =\sigma_{23}\sigma_{24}+(\sigma_{13}+\sigma_{34})(\sigma_{23}+\sigma_{24}) \end{array}.$$

We used Kagan’s developments [28] to further analyse the relationship between $\sigma_{\text{E}}$ and individual conductances $\sigma_{ij}$’s in a circuit. Equation (2.4) being the quotient of two affine functions is a hyperbola equation (unless C=0, in which case the function is strictly linear, but this could be obtained only by choosing ad hoc conductance values). Using the theory of concave functions and Jacobi’s theorem, we show in appendix A that the relationship between $\sigma_{ij}$ and $\sigma_{\text{E}}$ is necessarily concave for all $\sigma_{ij}$ and tends towards a horizontal asymptote with a value of A/C. Figure 1*d* shows the curves in the case of a Wheatstone bridge with arbitrary conductance values. Simulations of circuits with different topologies were carried out with LTSpice${,}^{\text{®}}$ [30] and gave consistent results: we observed in all cases increasing hyperbolae with horizontal asymptotes (not shown).

### 2.6. From electrical circuits to metabolic networks

The previous developments can be applied to metabolic networks of uni–uni reactions but are more laborious to write owing to the presence of equilibrium constants of the reactions that have no equivalent term in electrical circuits. However, since these additional parameters are necessarily constant and positive, they do not alter the structure of the equations and hence the conclusions drawn from them. To illustrate this point, we examined the case of a metabolic network with the same topology as a Wheatstone bridge (appendix B). The comparison of equations (2.5) (electrical circuit) and (B 4) (metabolic network) shows their structural equivalence. Therefore, in any network of uni–uni reactions catalysed by Michaelian enzymes that are far from saturation, the relationship between an enzyme parameter (kinetic parameter or concentration) and the flux is an increasing concave function with a horizontal asymptote (with the exception of the unrealistic case where C=0 (see above)).

Is this result valid for more general cases, i.e. for networks with both uni–uni and bi–bi reactions, which represent the majority of networks? In the case where two substrates A and B bind randomly to enzyme E, the reaction is:


E + A + B⇌EAB⇌E + P + Q,

where P and Q are the products. If the enzyme is far from saturation, the reaction rate is [31]:


(2.6) $$v\approx [E]\;\frac{k_{\text{cat}}}{K_{\text{M}}}\;\;\left(X_{\text{A}}X_{\text{B}}-\frac{X_{\text{P}}X_{\text{Q}}}{K_{\text{eq}}}\right),$$

where [E], $k_{\text{cat}}$ and $K_{\text{M}}$ are, respectively, the concentration, the catalytic constant and the Michaelis constant of the enzyme for both substrates, $X_{\text{A}}$, $X_{\text{B}}$, $X_{\text{P}}$ and $X_{\text{Q}}$ are, respectively, the concentration of substrates A and B and products P and Q, and $K_{\text{eq}}$ is the equilibrium constant of the reaction. In the graph of the network we can consider that A + B and P + Q are nodes. Since equation (2.6) has the same form as equation (2.2), the behaviour of diminishing returns applies.

The equations are more complex when substrates bind in a specific order, as well as when a ping-pong mechanism takes place where the enzyme is chemically and reversibly modified. So we were not able to prove formally the generality of the concave response. However, as any bi–bi reaction can be described as a linear series of elementary reactions involving the same enzyme, or two forms of an enzyme, it seems possible to assume that the response of the total flux to a change in enzyme efficiency is qualitatively similar to that observed with uni–uni reactions.

## 3. Discussion

Using an analogy with electrical circuits, we have been able to establish that the law of diminishing returns is valid for every enzyme in a metabolic network of uni–uni reactions, irrespective of network topology. This result is also valid for a type of bi–bi reaction; however, further developments and/or computer simulations are required for a full generalization.

The concavity of the enzyme–flux relationship is expected to increase with the number of enzymes in the network. Indeed, the summation property of the flux control coefficients states that $\sum_{k=1}^{n}C_{F_{k}}^{J}=\sum_{k=1}^{n}(\partial \;\ln J/\partial \;\ln F_{k})=1$, where n is the total number of enzymes [9,32]. Thus, the average control coefficient is 1/n: the more enzymes there are in the network, the smaller the control of the enzymes on the flux, on average. Smaller control means that enzyme efficiencies are at or near a plateau, which corresponds to a highly concave enzyme–flux relationship. This type of response may account in part for the selective neutrality of most molecular polymorphisms [33], the frequent epistasis between mutations [2,34] and the pervasive robustness within living systems [35–37].

Interestingly, several studies have reported that the robustness of gene expression patterns increases as the number of connections and regulatory factors increases (discussed in [38]). These results suggests a widespread link between robustness and network complexity, a link that is possibly valid for any network of transportation of matter and energy (metabolic networks, gene regulatory networks, signal transduction pathways, etc.). Thus, in addition to the numerous ‘local’ mechanisms of robustness that are assumed to result from natural selection (feedback loops, kinetic proofreading, modularity, redundancy, etc.; reviewed in [36,38–40]), there would be an intrinsic robustness, precluding any selective advantage, which emerges from the complexity of the global cellular network.

Finally, we now have a theoretical tool for evolutionary studies at our disposal to assess the effect of a mutation affecting any enzyme on the total flux of a system and to determine the evolutionary equilibria of enzyme concentrations or activities when the flux is under selective pressure, as Coton *et al*. [41,42] did for linear pathways.

## Data Availability

This article has no additional data.

## Appendix A. Relationship between the conductance of a resistor and the total current in an electrical circuit

### (a) Relationship between an individual conductance and the equivalent conductance

The junction equations for all node potentials in an electrical circuit of n nodes (n>2) can be expressed as:

ΣU=I,

where $𝐔=(U_{1},U_{2},\ldots ,U_{n}{)}^{T}$ is the vector of the potentials, $I=(I_{\text{out}},0,\ldots ,0,I_{\text{in}}{)}^{T}$ is the vector of the currents and Σ the conductance matrix in the electrical circuit,

$$\begin{array}{r} \Sigma =\left(\begin{array}{cccccc} c_{1} & -\sigma_{12} & -\sigma_{13} & -\sigma_{14} & \ldots & -\sigma_{1,n}\\ -\sigma_{21} & c_{2} & -\sigma_{23} & -\sigma_{24} & \ldots & -\sigma_{2,n}\\ -\sigma_{31} & -\sigma_{32} & c_{3} & -\sigma_{34} & \ldots & -\sigma_{3,n}\\ \vdots & \vdots & \vdots & \vdots & \ddots & \vdots \\ -\sigma_{n,1} & -\sigma_{n,2} & -\sigma_{n,3} & -\sigma_{n,4} & \ldots & c_{n} \end{array}\right) \end{array},$$

with $c_{i}=\sum_{\begin{array}{c} j\neq i\\ i=1 \end{array}}^{n}\sigma_{ij}$. Note that $\sigma_{ij}=0$ for non-connected nodes.

Kagan [28] derived the formula for the equivalent conductance in a generic non-simplifiable circuit and, by posing $U_{1}=\varepsilon$ and $U_{n}=0$, showed that

$$\sigma_{\text{E}}=\frac{\det\Sigma^{\prime }}{\det\Sigma^{\prime \prime }},$$

where $\Sigma^{\prime }$ is the upper left sub-matrix (n−1)×(n−1) of the conductance matrix Σ:

$$\begin{array}{r} \Sigma^{\prime }=\left(\begin{array}{cccccc} c_{1} & -\sigma_{12} & -\sigma_{13} & -\sigma_{14} & \ldots & -\sigma_{1,n-1}\\ -\sigma_{21} & c_{2} & -\sigma_{23} & -\sigma_{24} & \ldots & -\sigma_{2,n-1}\\ -\sigma_{31} & -\sigma_{32} & c_{3} & -\sigma_{34} & \ldots & -\sigma_{3,n-1}\\ \vdots & \vdots & \vdots & \vdots & \ddots & \vdots \\ -\sigma_{n-1,1} & -\sigma_{n-1,2} & -\sigma_{n-1,3} & -\sigma_{n-1,4} & \ldots & c_{n-1} \end{array}\right) \end{array},$$

and $\Sigma^{\prime \prime }$ is the lower right sub-matrix (n−2)×(n−2) of $\Sigma^{\prime }$:

$$\begin{array}{r} \Sigma^{\prime \prime }=\left(\begin{array}{ccccc} c_{2} & -\sigma_{23} & -\sigma_{24} & \ldots & -\sigma_{2,n-1}\\ -\sigma_{32} & c_{3} & -\sigma_{34} & \ldots & -\sigma_{3,n-1}\\ \vdots & \vdots & \vdots & \ddots & \vdots \\ -\sigma_{n-1,2} & -\sigma_{n-1,3} & -\sigma_{n-1,4} & \ldots & c_{n-1} \end{array}\right) \end{array}.$$

Kagan [28] showed that each term in $\det\;\Sigma^{\prime }$ and $\det\;\Sigma^{\prime \prime }$ is positive. Thus, the equivalent conductance is a ratio of two polynomials of degree (n−1) and (n−2), respectively, with only positive terms. Thus, it is possible to express $\sigma_{\text{E}}$ as:

(A 1)
$$\forall \sigma_{ij}\;\sigma_{\text{E}}=\frac{A\sigma_{ij}+B}{C\sigma_{ij}+D},$$

where A, B, C and D are non-negative terms that depend on all conductances other than $\sigma_{ij}$.

### (b) Proof of concavity

A real-valued function f:ℝ→ℝ is said to be concave if, ∀x,y∈ℝ and ∀α∈[0,1],

(A 2)
f((1−α)x+αy)≥(1−α)f(x)+αf(y).

The function f is given here by equation (A 1), so by substituting it in equation (A 2) we have:

$$\frac{((1-\alpha )\sigma_{ij}^{x}+\alpha \sigma_{ij}^{y})A+B}{((1-\alpha )\sigma_{ij}^{x}+\alpha \sigma_{ij}^{y})C+D}\geq (1-\alpha )\frac{\sigma_{ij}^{x}A+B}{\sigma_{ij}^{x}C+D}+\alpha \frac{\sigma_{ij}^{y}A+B}{\sigma_{ij}^{y}C+D}.$$

We have to prove that this inequality is valid ∀ij.

Taking the least common denominator and denoting $\sigma^{*}=((1-\alpha )\sigma_{ij}^{x}+\alpha \sigma_{ij}^{y})$, the above equation can be written as

(A 3)
$$\frac{\left(\sigma^{*}A+B)(\sigma_{ij}^{x}C+D)(\sigma_{ij}^{y}C+D\right)}{\left(\sigma^{*}C+D)(\sigma_{ij}^{x}C+D)(\sigma_{ij}^{y}C+D\right)}\;\geq \frac{\left(1-\alpha )(\sigma_{ij}^{x}A+B)(\sigma^{*}C+D)(\sigma_{ij}^{y}C+D)+\alpha (\sigma_{ij}^{y}A+B)(\sigma^{*}C+D)(\sigma_{ij}^{x}C+D\right)}{\left(\sigma^{*}C+D)(\sigma_{ij}^{x}C+D)(\sigma_{ij}^{y}C+D\right)}.$$

Given that $\forall \sigma_{ij}$, $\sigma_{ij}\geq 0$ and A,B,C,D≥0, equation (A 3) is satisfied if and only if

$$(\sigma^{*}A+B)(\sigma_{ij}^{x}C+D)(\sigma_{ij}^{y}C+D)\;\geq (1-\alpha )(\sigma_{ij}^{x}A+B)(\sigma^{*}C+D)(\sigma_{ij}^{y}C+D)+\alpha (\sigma_{ij}^{y}A+B)(\sigma^{*}C+D)(\sigma_{ij}^{x}C+D).$$

Thus,

$$\left(\sigma^{*}A+B)(\sigma_{ij}^{x}C+D)(\sigma_{ij}^{y}C+D)\;\geq (\sigma^{*}C+D)(AD\sigma^{*}+\sigma_{ij}^{x}\sigma_{ij}^{y}AC+((1-\alpha )\sigma_{ij}^{y}+\alpha \sigma_{ij}^{x})BC+BD\right)$$

or

(A 4)
$$ACD(\sigma^{*}-\sigma_{ij}^{y})(\sigma_{ij}^{x}-\sigma^{*})+BCD(\sigma_{ij}^{x}+\sigma_{ij}^{y}-\sigma^{*}-((1-\alpha )\sigma_{ij}^{y}+\alpha \sigma_{ij}^{x}))+BC^{2}(\sigma_{ij}^{x}\sigma_{ij}^{y}-\sigma^{*}((1-\alpha )\sigma_{ij}^{y}+\alpha \sigma_{ij}^{x}))\geq 0.$$

Noting that

$$(\sigma^{*}-\sigma_{ij}^{y})(\sigma_{ij}^{x}-\sigma^{*})=(\sqrt{\alpha (1-\alpha )}\sigma_{ij}^{x}-\sqrt{\alpha (1-\alpha )}\sigma_{ij}^{y}{)}^{2},$$

$$\sigma_{ij}^{x}+\sigma_{ij}^{y}-\sigma^{*}-((1-\alpha )\sigma_{ij}^{y}+\alpha \sigma_{ij}^{x})=0$$

and

$$\sigma_{ij}^{x}\sigma_{ij}^{y}-\sigma^{*}((1-\alpha )\sigma_{ij}^{y}+\alpha \sigma_{ij}^{x})=-(\sqrt{\alpha (1-\alpha )}\sigma_{ij}^{x}-\sqrt{\alpha (1-\alpha )}\sigma_{ij}^{y}{)}^{2}.$$

Equation (A 4) can be simplified to

(A 5)
AD−BC≥0.

Thus, this inequality must be satisfied to verify the concave relationship between a particular conductance, all others being constant, and the equivalent conductance.

Without loss of generality, we can focus on $\sigma_{12}$. We set $c_{1}^{\prime }=c_{1}-\sigma_{12}$, $c_{2}^{\prime }=c_{2}-\sigma_{12}$ and n−1=k.

So we have

$$\begin{array}{c} \begin{array}{c} A=\left|\begin{array}{cccc} c_{1}^{\prime }+c_{2}^{\prime } & -(\sigma_{13}+\sigma_{23}) & \ldots & -(\sigma_{1,k}+\sigma_{2,k})\\ -(\sigma_{31}+\sigma_{32}) & c_{3} & \ldots & -\sigma_{3,k}\\ \vdots \; & \vdots \; & \ddots & \vdots \;\\ -(\sigma_{k,1}+\sigma_{k,2}) & -\sigma_{k,3} & \ldots & c_{k} \end{array}\right| \end{array} \end{array}$$

$$\begin{array}{c} \begin{array}{c} B=\left|\begin{array}{ccccc} c_{1}^{\prime } & c_{1}^{\prime } & -\sigma_{13} & \ldots & -\sigma_{1,k}\\ c_{1}^{\prime } & c_{1}^{\prime }+c_{2}^{\prime } & -(\sigma_{13}+\sigma_{23}) & \ldots & -(\sigma_{1,k}+\sigma_{2,k})\\ -\sigma_{31} & -(\sigma_{31}+\sigma_{32}) & c_{3} & \ldots & -\sigma_{3,k}\\ \vdots \; & \vdots \; & \vdots \; & \ddots & \vdots \;\\ -\sigma_{k,1} & -(\sigma_{k,1}+\sigma_{k,2}) & -\sigma_{k,3} & \ldots & c_{k} \end{array}\right| \end{array} \end{array}$$

$$\begin{array}{c} \begin{array}{c} C=\left|\begin{array}{ccc} c_{3} & \ldots & -\sigma_{3,k}\\ \vdots \; & \ddots & \vdots \;\\ -\sigma_{k,3} & \ldots & -c_{k} \end{array}\right| \end{array} \end{array}$$

$$\begin{array}{r} D=\left|\begin{array}{cccc} c_{2}^{\prime } & -\sigma_{23} & \ldots & -\sigma_{2,k}\\ -\sigma_{32} & c_{3} & \ldots & -\sigma_{3,k}\\ \vdots & \vdots & \ddots & \vdots \\ -\sigma_{k,2} & -\sigma_{k,3} & \ldots & c_{k} \end{array}\right| \end{array}.$$

Now, we need to verify equation (A 5) to demonstrate the concave relationship between a particular conductance, all others being fixed, and the equivalent conductance. To this end, we used the Jacobi’s theorem.

Let M denotes the determinant of a matrix $M=\|m_{ij}{\|}_{1}^{n}$, $M^{c}$ denotes the determinant of the matrix of its cofactors $M^{c}=\|M_{ij}{\|}_{1}^{n}$, 1≤p<n and σ(i1…inj1…jn) denotes an arbitrary permutation of the n rows and columns of M. Then

$$\begin{array}{r} \left|\begin{array}{ccc} M_{i_{1}j_{1}} & \ldots & M_{i_{1}j_{p}}\\ M_{i_{2}j_{1}} & \ldots & M_{i_{2}j_{p}}\\ \vdots & \ddots & \vdots \\ M_{i_{p}j_{1}} & \ldots & M_{i_{p}j_{p}} \end{array}\right|=(-1{)}^{\sigma }\;\left|\begin{array}{ccc} m_{i_{p+1}j_{p+1}} & \ldots & m_{i_{p+1}j_{n}}\\ m_{i_{p+2}j_{p+1}} & \ldots & m_{i_{p+2}j_{n}}\\ \vdots & \ddots & \vdots \\ m_{i_{n}j_{p+1}} & \ldots & m_{i_{n}j_{n}} \end{array}\right|\cdot M^{p-1} \end{array}.$$

Now, note that C is a minor of B obtained by deleting the first two rows and columns of B. Using Jacobi’s theorem, with p=2, n=k, $i_{l}=l$ and $j_{l}=l$ ∀l∈1,…k, we can express BC as

(A 6)
$$\begin{array}{r} \left|\begin{array}{cc} B_{11} & B_{12}\\ B_{21} & B_{22} \end{array}\right|=(-1{)}^{\sigma }CB^{2-1}=CB \end{array},$$

since σ=0 (there are no permutations).

Using the sum property of the determinants, we can rewrite B as

$$\begin{array}{r} B=\left|\begin{array}{ccccc} c_{1}^{\prime } & 0 & -\sigma_{13} & \ldots & -\sigma_{1,k}\\ 0 & c_{2}^{\prime } & -\sigma_{23} & \ldots & -\sigma_{2,k}\\ -\sigma_{31} & -\sigma_{32} & c_{3} & \ldots & -\sigma_{3,k}\\ \vdots & \vdots & \ddots & \vdots \\ -\sigma_{k,1} & -\sigma_{k,2} & -\sigma_{k,3} & \ldots & c_{k} \end{array}\right| \end{array},$$

and give an explicit expression of the cofactors in equation (A 6):

$$\begin{array}{c} B_{11}=D \end{array}$$

$$\begin{array}{c} \begin{array}{c} B_{12}=-\left|\begin{array}{cccc} 0 & -\sigma_{23} & \ldots & -\sigma_{2,k}\\ -\sigma_{31} & c_{3} & \ldots & -\sigma_{3,k}\\ \vdots \; & \vdots \; & \ddots & \vdots \;\\ -\sigma_{k,1} & -\sigma_{k,3} & \ldots & c_{k} \end{array}\right|=-B^{**} \end{array} \end{array}$$

$$\begin{array}{c} \begin{array}{c} B_{21}=-\left|\begin{array}{cccc} 0 & -\sigma_{13} & \ldots & -\sigma_{1,k}\\ -\sigma_{32} & c_{3} & \ldots & -\sigma_{3,k}\\ \vdots \; & \vdots \; & \ddots & \vdots \;\\ -\sigma_{k,2} & -\sigma_{k,3} & \ldots & c_{k} \end{array}\right|=-B^{*} \end{array} \end{array}$$

$$\begin{array}{r} B_{22}=\left|\begin{array}{cccc} c_{1}^{\prime } & -\sigma_{13} & \ldots & -\sigma_{1,k}\\ -\sigma_{31} & c_{3} & \ldots & -\sigma_{3,k}\\ \vdots & \vdots & \ddots & \vdots \\ -\sigma_{k,1} & -\sigma_{k,3} & \ldots & c_{k} \end{array}\right| \end{array}.$$

Thus,

$$\begin{array}{r} BC=\left|\begin{array}{cc} D & -B^{**}\\ -B^{*} & B_{22} \end{array}\right|=DB_{22}-(B^{*}{)}^{2} \end{array},$$

where $B^{*}=B^{**}$ since the respective matrices are a transpose of each other.

On the other hand, we can rewrite A as a function of D, $B^{*}$ and $B_{22}$. Using the properties of determinants, we get

$$A=2B^{*}+D+B_{22}.$$

We can now substitute all this information in equation (A 5):

$$AD-BC=(2B^{*}+D+B_{22})D-(B_{22}D-(B^{*}{)}^{2})=2B^{*}D+D^{2}+(B^{*}{)}^{2}=(D+B^{*}{)}^{2},$$

which is always equal to or greater than zero. Thus, equation (A 5) is always satisfied, meaning that the relationship between any conductance and the equivalent conductance is concave for any electrical circuit, the limits of the hyperbola being

$$\lim_{\sigma_{ij}\to 0}\sigma_{\text{E}}=B/D\;\text{and}\;\lim_{\sigma_{ij}\to +\infty }\sigma_{\text{E}}=A/C.$$

As the equivalent conductance is proportional to the total current through the circuit, we have shown that the law of diminishing returns applies to the relationship between any conductance and the total current, irrespective of the topology of the circuit.

## Appendix B. Equivalent enzyme efficiency in the case of a Wheatstone-type metabolic network

Adapting Kagan’s formalism [28] to metabolic networks requires the introduction of the equilibrium constants into the developments. As an example, we give below the expression of the equivalent enzyme efficiency $F_{E}$ for a Wheatstone-type metabolic network (figure 1*b* in the main text).

The product of the reaction $X_{P}$ is here $X_{4}$. The rate of production of $X_{4}$ is:

$$\frac{\text{d}X_{4}}{\text{d}t}=J=v_{2}+v_{5}.$$

Replacing J, $v_{2}$ and $v_{5}$ by their expression, we get:

$$J=F_{E}\;(X_{1}-X_{4}/K_{E})=F_{2}\;(X_{2}-X_{4}/K_{4})+F_{5}\;(X_{3}-X_{4}K_{4}).$$

Assuming $X_{4}=0$, the flux writes:

$$J=X_{2}F_{2}+X_{3}F_{5}=F_{E}X_{1}.$$

Therefore,

(B 1)
$$F_{E}=\frac{X_{2}F_{2}+X_{3}F_{5}}{X_{1}}.$$

We have now to determine the expressions of $X_{2}$ and $X_{3}$. The rates of production of $X_{2}$ and $X_{3}$ write:

$$\begin{array}{rl} \frac{\text{d}X_{2}}{\text{d}t}=0 & =-v_{3}-v_{2}+v_{1}\\ & =-F_{3}(X_{2}-X_{3}/K_{3})-F_{2}(X_{2}-X_{4}/K_{2})+F_{1}(X_{1}-X_{2}/K_{1})\\ & =X_{1}F_{1}-X_{2}(F_{1}/K_{1}+F_{2}+F_{3})+X_{3}F_{3}/K_{3}+X_{4}F_{2}/K_{2}\\ \frac{\text{d}X_{3}}{\text{d}t}=0 & =v_{4}+v_{3}-v_{5}\\ & =F_{4}(X_{1}-X_{3}/K_{4})+F_{3}(X_{2}-X_{3}/K_{3})-F_{5}(X_{3}-X_{4}/K_{5})\\ & =X_{1}F_{4}+X_{2}F_{3}-X_{3}(F_{3}/K_{3}+F_{4}/K_{4}+F_{5})+X_{4}F_{5}/K_{5} \end{array}.$$

Therefore, because $X_{4}=0$:

(B 2)
$$\begin{array}{rl} X_{2}c_{2} & =X_{1}F_{1}+X_{3}F_{3}/K_{3}, \end{array}$$

(B 3)
$$\begin{array}{rl} X_{3}c_{3} & =X_{1}F_{4}+X_{2}F_{3}, \end{array}$$

where $c_{2}=F_{1}/K_{1}+F_{2}+F_{3}$ and $c_{3}=F_{3}/K_{3}+F_{4}/K_{4}+F_{5}$. Introducing $X_{3}$ equation (B 3) into equation (B 2), we get:

$$\begin{array}{rl} X_{2}c_{2} & =X_{1}F_{1}+\frac{X_{1}F_{4}+X_{2}F_{3}}{c_{3}}F_{3}/K_{3}\\ X_{2} & =X_{1}\frac{F_{1}c_{3}K_{3}+F_{3}F_{4}}{c_{2}c_{3}K_{3}-F_{3}^{2}} \end{array},$$

and introducing $X_{2}$ equation (B 2) into equation (B 3) we get:

$$\begin{array}{rl} X_{3}c_{3} & =X_{1}F_{4}+\frac{X_{1}F_{1}+X_{3}F_{3}/K_{3}}{c_{2}}F_{3}\\ X_{3} & =X_{1}\frac{F_{4}c_{2}K_{3}+F_{1}F_{3}K_{3}}{c_{3}c_{2}K_{3}-F_{3}^{2}} \end{array}.$$

Replacing $X_{2}$ and $X_{3}$ in equation (B 1) by their expression, we get:

$$\begin{array}{rl} F_{E} & =\frac{X_{2}F_{2}}{X_{1}}+\frac{X_{3}F_{5}}{X_{1}}\\ & =\frac{F_{2}(F_{1}c_{3}K_{3}+F_{3}F_{4})+F_{5}(F_{4}c_{2}K_{3}+F_{1}F_{3}K_{3})}{c_{2}c_{3}K_{3}-F_{3}^{2}} \end{array},$$

and replacing $c_{2}$ and $c_{3}$ by their expression, we obtain the equivalent enzyme efficiency of a Wheatstone-type metabolic network:

(B 4)
$$F_{E}=\frac{F_{1}F_{2}K_{3}(F_{3}/K_{3}+F_{4}/K_{4}+F_{5})+F_{2}F_{3}F_{4}+F_{1}F_{3}F_{5}K_{3}+F_{4}F_{5}K_{3}(F_{1}/K_{1}+F_{2}+F_{3})}{F_{3}(F_{1}/K_{1}+F_{2}+F_{4}K_{3}/K_{4}+F_{5}K_{3})+K_{3}(F_{1}/K_{1}+F_{2})(F_{4}/K_{4}+F_{5})}.$$

This relationship has the same structure as that of equation (2.5), except that it includes the equilibrium constants $K_{i}$, which are strictly positive. Therefore, the relationship between the equivalent enzyme efficiency, which is proportional to the flux, and any particular enzyme efficiency, is concave with a horizontal asymptote.
